# Naturally Occurring PB2 Paired Substitutions Confer Host-Dependent Polymerase Phenotypes in H7N9 Avian Influenza Viruses with Differential ANP32 Support

**DOI:** 10.3390/ani16213535

**Published:** 2026-11-09

**Authors:** Zhuxing Ji, Yu Han, Xiaolong Lu, Wenhao Yang, Ruyi Gao, Yu Chen, Min Gu, Jiao Hu, Xiaowen Liu, Shunlin Hu, Xiufan Liu, Xiaoquan Wang, Kaituo Liu

**Affiliations:** 1 Key Laboratory of Avian Bioproducts Development, Ministry of Agriculture and Rural Affairs, College of Veterinary Medicine, Yangzhou University, Yangzhou 225009, China; 2 Joint International Research Laboratory of Agriculture and Agri-Product Safety, The Ministry of Education of China, Yangzhou University, Yangzhou 225009, China; 3 Jiangsu Co-Innovation Center for Prevention and Control of Important Animal Infectious Diseases and Zoonosis, Yangzhou University, Yangzhou 225009, China; 4 Jiangsu Key Laboratory of Zoonosis, Yangzhou 225009, China

**Keywords:** H7N9, PB2, paired substitution, polymerase activity, ANP32

## Abstract

H7N9 avian influenza viruses have caused repeated human infections since 2013, but the effects of naturally occurring PB2 substitution combinations on polymerase activity remain poorly understood. We analyzed 899 avian-derived and 1087 human-derived H7N9 PB2 sequences collected between 2013 and 2025. Six PB2 positions (109, 122, 191, 340, 559, and 640) showed distinct frequency changes across epidemic waves and host origins. Pairwise analysis identified five representative combinations with different temporal patterns. In minigenome assays, four paired substitutions reduced polymerase activity in human 293T cells at both 33 °C and 37 °C, whereas none of the five significantly altered activity in chicken DF1 cells at 37 °C or 41 °C. Several paired substitutions showed polymerase activity profiles different from those of the corresponding single substitutions in the tested cellular assays. ANP32 overexpression experiments showed different polymerase activity responses across the tested cellular conditions. Under chicken ANP32A expression, none of the paired-substitution polymerases differed significantly from the wild type, whereas human ANP32A and ANP32B overexpression did not enhance their activity in DF1 cells. Co-immunoprecipitation confirmed detectable interactions between PB2 variants and human or chicken ANP32 proteins. Structural modeling further revealed differences in predicted PB2–ANP32A interfaces among the tested variants. Together, these results show that the tested PB2 substitution combinations produced different polymerase activity profiles in human 293T and chicken DF1 cells and exhibited different responses to ANP32 overexpression under the examined conditions. The findings provide insight into the functional consequences of PB2 evolution during H7N9 circulation.

## Simple Summary

H7N9 avian influenza viruses circulate in poultry and can infect humans. Changes in the viral PB2 protein can affect how efficiently the virus copies its genetic material, but the effects of naturally occurring combinations of these changes remain unclear. We analyzed H7N9 PB2 sequences collected from birds and humans between 2013 and 2025 and identified several recurring combinations with different patterns over time. Laboratory experiments showed that four combinations reduced polymerase activity in human cells but caused no significant changes in chicken cells. Individual changes did not always produce the same effects when combined. ANP32 overexpression produced different polymerase activity responses under the tested cellular conditions. These findings show that the tested PB2 combinations can affect polymerase reporter activity differently in human 293T and chicken DF1 cells.

## 1. Introduction

H7N9 avian influenza viruses, which belong to the ICTV species *Alphainfluenzavirus influenzae*, were first recognized as a cause of human infection in China in 2013 [1]. Between 2013 and 2017, the virus caused five epidemic waves, with the fifth wave reaching the largest scale of the series [2]. According to the World Health Organization (WHO), 1568 laboratory-confirmed human infections with influenza A(H7N9), including 616 fatal cases, have been reported since early 2013 [3]. The epidemiological landscape changed markedly after implementation of the national H7 poultry vaccination program, which was followed by a sharp decline in H7N9 circulation and human infections [4]. The end of large-scale human epidemics did not mark the end of H7N9 evolution in poultry. Viruses detected after vaccine implementation retained substantial genetic and biological diversity [5], and sequence analyses identified accelerated evolutionary change during the vaccination era [6]. Surveillance across the following decade further documented continuing genetic evolution and antigenic differentiation [7]. The post-wave-5 period therefore represents a distinct phase of H7N9 evolution in which viral genetic backgrounds continued to change under a substantially altered poultry immune and epidemiological environment. Identifying which polymerase backgrounds became recurrent during this period can complement analyses of individual substitutions.

PB2 is a major determinant of influenza polymerase performance and host range. H7N9 studies have established that substitutions in this subunit can substantially alter polymerase activity and mammalian phenotypes [8]. The canonical E627K substitution illustrates how rapidly PB2 states can change during adaptation: within-host analysis of H7N9 documented dynamic PB2-E627K substitution during infection [9]. Large-scale mutational mapping has further shown that the adaptive landscape of PB2 extends well beyond a small set of canonical residues [10]. More importantly, the phenotype of one substitution depends on the surrounding polymerase background. Epistatic interactions within the influenza polymerase complex can redirect the effects of individual changes [11], and H7N9-specific work has shown that PB2-V292I can modify the effect of E627K on polymerase activity and replication [12]. Our previous studies reached the same conclusion at the viral-genome level: two closely related H7N9 isolates with only a small number of amino acid differences displayed markedly different pathogenic phenotypes [13], and PB2-E627K did not reproduce the full phenotype unless it occurred in the appropriate PB1/PA background [14]. These observations make naturally occurring PB2 combinations a biologically meaningful unit for functional analysis.

Host proteins provide an additional layer of context for PB2 function. ANP32 family proteins are central cofactors of the influenza polymerase, and species-specific differences in ANP32A form a molecular basis for restriction of avian polymerases in mammalian cells [15]. Human ANP32A and ANP32B are functionally redundant host factors that support influenza virus replication in human cells [16]. Structural work places ANP32 directly within the replication machinery, where ANP32A supports assembly of an asymmetric polymerase replicase complex [17]. ANP32 identity can also shape the evolutionary routes available to the polymerase, because mammalian ANP32A and ANP32B drive distinct polymerase adaptations [18]. PB2 phenotype is therefore determined by an interaction between viral sequence background and the host-factor environment. This relationship is especially relevant to recurrent PB2 combinations that become enriched in avian viruses but display different activities in mammalian and avian cellular backgrounds.

In this study, we characterized wave-stratified amino acid frequencies at six selected PB2 positions in avian- and human-derived H7N9 sequences and examined the co-occurrence of their residue states. Five representative naturally occurring paired backgrounds were chosen for functional analysis. The corresponding single and paired substitutions were introduced into the same early-wave H7N9 polymerase background, and their effects on polymerase activity were compared in human 293T and chicken DF1 cells. We further examined responses to human and chicken ANP32 overexpression using minigenome assays, co-immunoprecipitation, and structural modeling.

## 2. Materials and Methods

### 2.1. Sequence Datasets and Epidemic Wave Definitions

Publicly available PB2 sequences of H7N9 influenza A viruses sampled between 2013 and 2025 were obtained from the GISAID EpiFlu database, which was last accessed on 30 December 2025. PB2 sequences with available host metadata were included in the curated dataset. Sequences containing partial PB2 regions or ambiguous nucleotide calls were retained where applicable, and unresolved amino acid states were handled on a position-specific basis, as described in Section 2.3 and Section 2.4. The initial curated sequence set comprised 899 avian-derived and 1091 human-derived PB2 records, as defined by host metadata in GISAID. During quality control, records with duplicated accession numbers were identified, and only one record per accession number was retained. After duplicate-accession removal, the frequency-analysis dataset comprised 899 avian-derived and 1087 human-derived PB2 records. PB2 nucleotide sequences were aligned using the ClustalW algorithm implemented in MEGA 7.

Epidemic waves were assigned according to the sampling date. Wave 1 (W1) was defined as February 2013 to September 2013; wave 2 (W2), October 2013 to September 2014; wave 3 (W3), October 2014 to September 2015; wave 4 (W4), October 2015 to September 2016; wave 5 (W5), October 2016 to September 2017; and after wave 5 (after W5), October 2017 to December 2025. The post-wave-5 human-derived dataset contained 11 sequences and was temporally uneven, comprising one sequence from 2017, two from 2018, and eight from 2019, with no human-derived sequences available in the analyzed dataset from 2020 to 2025.

### 2.2. Site Prioritization for PB2 Sequence Analysis

PB2 amino acid alignments from avian- and human-derived H7N9 sequences were examined across epidemic waves to characterize changes in residue-state frequencies. Six PB2 positions (109, 122, 191, 340, 559, and 640) were selected for exploratory analysis based on their distinct temporal patterns. The frequencies of 191E and 559T increased during the early waves, those of 109I, 122A, and 640I increased in later waves, and 340K reached its highest frequency in wave 5.

All 15 pairwise combinations of the six selected residue states were analyzed descriptively across host groups and epidemic waves. Five combinations were selected for functional analysis to represent two contrasting temporal patterns in avian-derived sequences: four combinations that became more prevalent in later waves and after wave 5, and one combination that peaked in wave 5. Together, these five combinations covered all six selected PB2 positions.

### 2.3. Extraction of PB2 Amino Acid States

**Figure 1:**
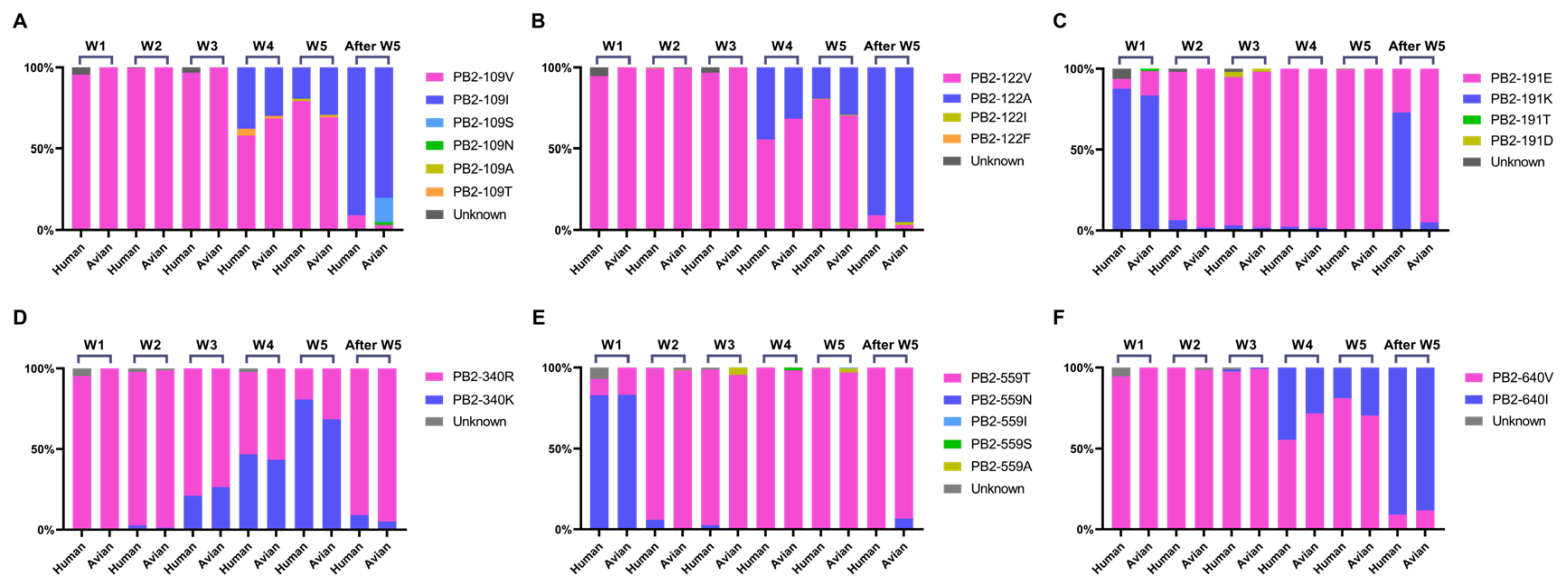
Wave- and host-associated frequency changes at six PB2 positions in avian- and human-derived H7N9 viruses. Frequencies of the indicated residue states at PB2 positions 109 (**A**), 122 (**B**), 191 (**C**), 340 (**D**), 559 (**E**), and 640 (**F**) across epidemic waves (W1–W5) and after wave 5. For each wave and host category, percentages were calculated using the total number of sequences in that host–wave group as the denominator. Residues that could not be unambiguously assigned at the indicated PB2 position were shown as Unknown. Avian-derived and human-derived datasets are shown together within each panel for direct comparison. Only publicly available PB2 sequences deposited in GISAID were included. The database was last accessed on December 2025.

Six PB2 amino acid positions, 109, 122, 191, 340, 559, and 640, were analyzed in this study. Amino acid states at these positions were extracted from each PB2 sequence and compiled into wave- and host-stratified tables. For single-position frequency analysis in Figure 1, percentages were calculated using the total number of sequences in each host–wave group as the denominator. Residues that could not be unambiguously assigned at the indicated PB2 position were shown as Unknown.

### 2.4. Pairwise Prevalence Calculation

For each epidemic wave and host group, the prevalence of each PB2 paired background was calculated using a pair-specific denominator. For a given pair of PB2 positions, sequences were included in the denominator only when both relevant positions had unambiguous amino acid calls. Sequences containing missing, ambiguous, or blank calls at either relevant position were excluded from the denominator for that paired background. The numerator was defined as the number of sequences carrying both target residues at the two specified positions. Pairwise prevalence was therefore calculated as x/n_pair × 100%, where x indicates the number of sequences carrying both target residues and n_pair indicates the number of sequences with unambiguous amino acid calls at both relevant positions.

### 2.5. Cells

Human embryonic kidney 293T cells and chicken embryo fibroblast DF1 cells were obtained from the American Type Culture Collection (ATCC). Both cell lines were maintained in Dulbecco’s modified Eagle’s medium (DMEM; Gibco, Thermo Fisher Scientific, Cat. No. 21068028) supplemented with 10% fetal bovine serum (FBS; EallBio Life Sciences, Cat. No. 03.A16001DC) at 37 °C in a humidified incubator with 5% CO_2_.

### 2.6. Plasmids and PB2 Mutational Combinations

The H7N9 strain A/chicken/Eastern China/JTC4/2013 [13,14] was used as the uniform early-wave polymerase background for functional analyses. The parental PB2 sequence of A/chicken/Eastern China/JTC4/2013 contained glutamic acid (E) at position 627 and aspartic acid (D) at position 701. Expression plasmids encoding PB2, PB1, PA, and NP were used for minigenome polymerase activity assays. PB2 variants carrying the indicated single substitutions, V109I, V122A, K191E, R340K, N559T, and V640I, or paired substitutions, V109I + K191E, K191E + V640I, V109I + V640I, K191E + R340K, and V122A + N559T, were generated by site-directed mutagenesis and confirmed by sequencing.

Human ANP32A, human ANP32B, chicken ANP32A, and chicken ANP32B expression plasmids were used for ANP32 overexpression and co-immunoprecipitation assays. These ANP32 expression constructs were based on the pCAGGS vector. Epitope-tagged ANP32 constructs were used for immunoblotting and immunoprecipitation, and tag-specific antibodies were used as appropriate.

### 2.7. Minigenome Polymerase Activity Assays

Minigenome assays were performed to measure influenza polymerase activity [14]. Briefly, 293T or DF1 cells cultured in 12-well plates were co-transfected with expression plasmids encoding PB2, PB1, PA, and NP, an influenza virus-like firefly luciferase reporter plasmid, and a Renilla luciferase plasmid using PolyFect Transfection Reagent (QIAGEN, Cat. No. 301105; 5 μL per well), according to the manufacturer’s instructions. At 24 h post-transfection, cells were lysed with CLT lysis buffer (200 μL per well), and luciferase activities were measured using a dual-luciferase assay system (Vazyme, Nanjing, China, Cat. No. DL101-01). Firefly luciferase activity was normalized to Renilla luciferase activity, and polymerase activity was expressed relative to the indicated control group.

For paired-substitution assays, 293T cells were incubated at 33 °C or 37 °C, and DF1 cells at 37 °C or 41 °C. Polymerase activity was expressed relative to WT under the same cell and temperature conditions. For single-substitution assays, polymerase activity was measured in 293T cells at 33 °C and 37 °C and expressed relative to WT at the corresponding temperature.

### 2.8. Effects of ANP32 Overexpression on Polymerase Activity

For ANP32 overexpression assays in 293T cells, expression plasmids encoding huANP32A, huANP32B, chANP32A, or chANP32B were co-transfected with the minigenome system containing WT or PB2 paired-substitution polymerases. Polymerase activity was measured at 37 °C. For analyses comparing ANP32-mediated enhancement within each PB2 background, polymerase activity was expressed relative to the corresponding empty-vector control. For comparisons among WT and paired-substitution polymerases under huANP32A or chANP32A expression, polymerase activity was expressed relative to WT under the corresponding ANP32A condition.

For DF1 ANP32 expression assays, huANP32A or huANP32B was co-expressed with each PB2 paired-substitution polymerase in the minigenome system. Polymerase activity was measured at 37 °C and expressed relative to the corresponding empty-vector control.

### 2.9. Western Blot Analysis

Western blotting was performed to assess the expression of viral PB2 and tagged ANP32 proteins under the corresponding experimental conditions. Cells were harvested 24 h after transfection and lysed in cell lysis buffer containing protease inhibitors. Protein samples were mixed with SDS-PAGE loading buffer, denatured, separated by SDS-PAGE, and transferred onto PVDF membranes. After blocking with 5% BSA, membranes were incubated with primary antibodies against PB2 (GenTex, Cat. No. GTX125926), His-tag (Beyotime, Cat. No. AB3071), Flag-tag (Beyotime, Cat. No. AB3055), or tubulin (Beyotime, Cat. No. AF1216), as appropriate. A prestained protein ladder (Beyotime, Cat. No. P0078) was used to estimate molecular weights. Membranes were then washed and incubated with HRP-conjugated goat anti-mouse or goat anti-rabbit IgG secondary antibodies (CWBIO, Beijing, China; Cat. Nos. CW0103S and CW0102S). Protein bands were visualized using enhanced chemiluminescence (ECL; Beyotime, Cat. No. P0018HS).

### 2.10. Co-Immunoprecipitation

To assess detectable ANP32–PB2 association by co-immunoprecipitation (Co-IP), 293T cells were co-transfected with plasmids expressing tagged huANP32A, huANP32B, chANP32A, or chANP32B and plasmids expressing WT PB2 or the indicated paired-substitution variants. Cells were lysed 24 h after transfection in immunoprecipitation lysis buffer containing protease inhibitors. A portion of each lysate was retained as the input sample.

Tagged proteins were immunoprecipitated using a Protein A + G Magnetic Beads Immunoprecipitation Kit (Beyotime, Shanghai, China, Cat. No. P2179S). Briefly, 8 μL of magnetic beads and 10 μL of specific antibodies were added to the cell lysates and incubated overnight at 4 °C with rotation, according to the manufacturer’s instructions. Input and immunoprecipitated fractions were analyzed by Western blotting. Associated PB2 was detected using an anti-PB2 antibody, and β-tubulin served as the loading control for input samples.

### 2.11. Structural Modeling and Interface Analysis

Structural modeling was performed using WT PB2 and two representative paired mutants (V109I + K191E and V122A + N559T) with human ANP32A (huANP32A) and chicken ANP32A (chANP32A). PB2 structures were predicted using AlphaFold 3, and the top-ranked models were selected for analysis. The crystal structure of huANP32A (UniProt: P39687; PDB: 4XOS) was obtained from the Protein Data Bank. The chANP32A structure was predicted using AlphaFold 3 based on its amino acid sequence (NCBI: XP_046755145.1).

Protein–protein docking was performed using HDOCKlite v1.1 [19,20] without predefined binding sites. The top-ranked docking model (model_1) was selected for each complex, and docking scores and confidence scores were recorded. Binding free energies were estimated using the MM/GBSA method through the HawkDock server under its default computational settings [21].

Protein–protein interfaces were analyzed using PLIP [22] to identify hydrogen bonds, salt bridges, hydrophobic contacts, and π-related interactions. Hydrophobic contacts were defined by an interatomic distance of ≤4.0 Å, hydrogen bonds by a donor–acceptor distance of ≤4.1 Å and a donor angle of ≥100°, and salt bridges by a distance of ≤5.5 Å between oppositely charged centers. PyMOL (version 2.5) was used to visualize the predicted complexes and their interactions.

### 2.12. Statistical Analysis

Statistical analyses were performed using GraphPad Prism (version 8). Data are presented as mean ± standard deviation (SD) from three independent biological replicates per condition (n = 3), unless otherwise indicated. Polymerase activity was compared within the same cell type, temperature, and experimental condition. Differences among groups were analyzed using ordinary one-way analysis of variance (ANOVA), followed by Dunnett’s multiple-comparisons test against the indicated control group. A *p* value < 0.05 was considered statistically significant. Significance levels are indicated in the figure legends.

## 3. Results

### 3.1. Six PB2 Positions Show Wave- and Host-Associated Frequency Shifts During H7N9 Circulation

To examine changes in PB2 amino acid frequencies during H7N9 circulation, we analyzed 899 avian-derived and 1087 human-derived PB2 sequences collected between 2013 and 2025 (Supplementary Table S1). Six positions (109, 122, 191, 340, 559, and 640) were selected for analysis based on their frequency patterns across the six sampling periods and host groups (Figure 1A–F). From wave 1 to wave 5, frequency trajectories at these positions were broadly concordant between avian- and human-derived sequences. The 191E residue (detected as early as wave 1 in avian-derived sequences) and 559T increased sharply between waves 1 and 2 and remained at high frequencies through wave 5 in both datasets. In contrast, 109I, 122A, and 640I were absent or rare during waves 1–3 and increased in frequency from wave 4 onward. The 340K residue increased progressively through waves 3–5 in both host groups before declining after wave 5.

After wave 5, 109I, 122A, 559T, and 640I were predominant in both avian- and human-derived sequences, whereas 340K was uncommon in both datasets (Figure 1A–F; Supplementary Table S1). A notable difference between host datasets occurred at position 191: 191E remained at high frequency in avian-derived sequences (57/60, 95.0%) but was detected in only 3/11 (27.3%) human-derived sequences. These 11 post-wave-5 human-derived sequences comprised one sequence from 2017, two from 2018, and eight from 2019, with no human-derived sequences from 2020 to 2025 in the analyzed dataset. All three sequences from 2017–2018 carried 191E, whereas all eight sequences from 2019 carried 191K; thus, the lower aggregate frequency of 191E in the post-wave-5 human dataset reflects the predominance of 191K in the 2019 samples.

Overall, the six selected PB2 positions showed distinct temporal frequency patterns during H7N9 circulation and were subsequently examined for pairwise amino acid co-occurrence.

### 3.2. Pairwise Analysis Identifies Five Representative PB2 Paired Backgrounds with Distinct Temporal Patterns in Avian and Human Viruses

**Figure 2:**
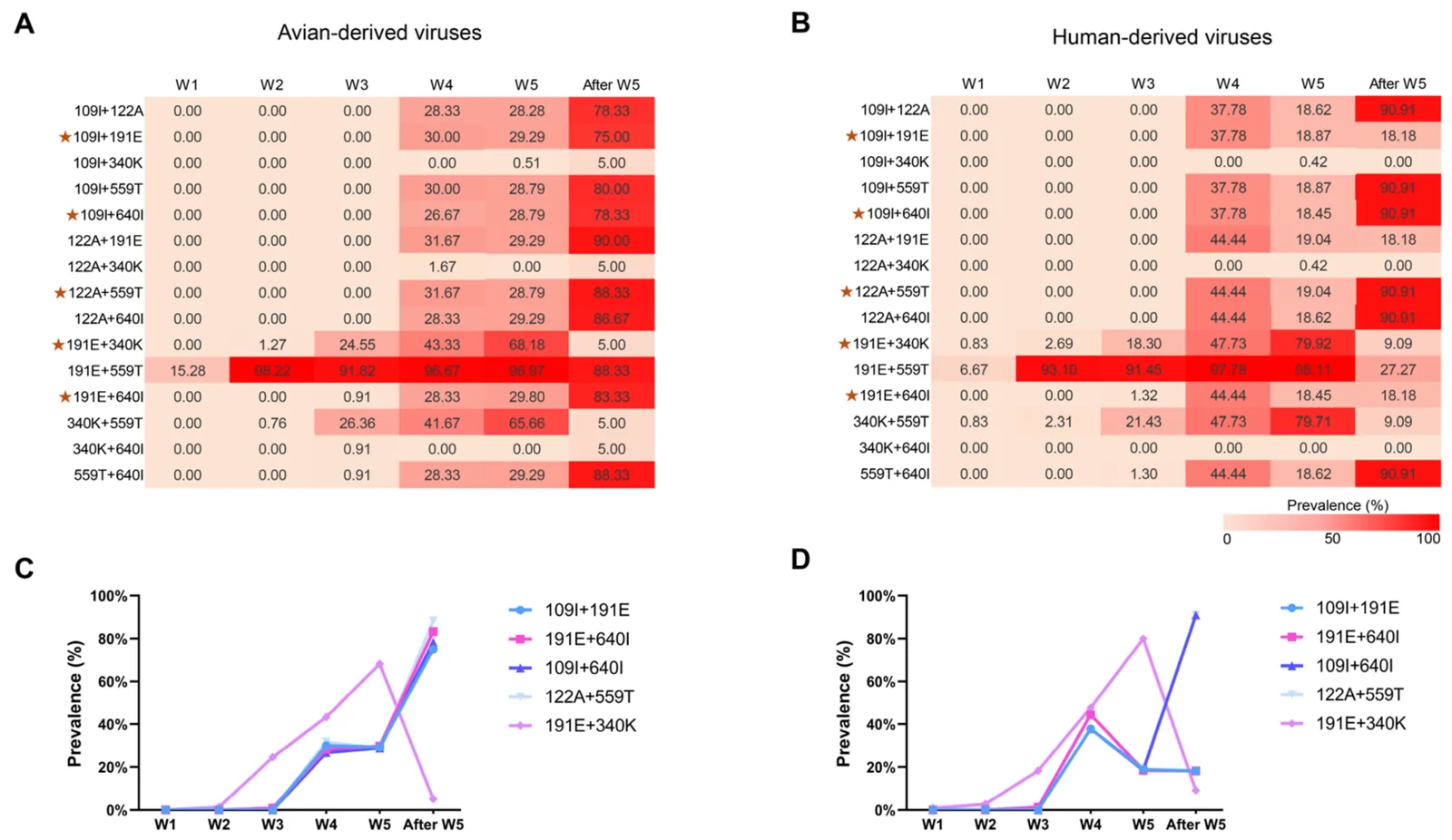
Pairwise analysis identifies PB2 paired backgrounds with distinct temporal patterns in avian- and human-derived H7N9 viruses. Heatmaps showing the prevalence of all 15 possible pairwise combinations among PB2 positions 109, 122, 191, 340, 559, and 640 in avian-derived (**A**) and human-derived (**B**) H7N9 viruses across epidemic waves. (**C**,**D**) Temporal trajectories of the five representative paired backgrounds selected for downstream functional analysis in avian-derived (**C**) and human-derived (**D**) viruses. Four pairs—109I + 191E, 191E + 640I, 109I + 640I, and 122A + 559T—showed a late-wave/post-wave-5 enriched pattern in avian viruses, whereas 191E + 340K showed a wave-5-peaking pattern followed by decline. Pairwise prevalence was calculated using pair-specific denominators; sequences with missing, ambiguous, or blank calls at either relevant PB2 position were excluded from n_pair. Detailed x/n_pair values are provided in Supplementary Tables S2 and S3. The post-wave-5 human-derived dataset comprised 11 sequences. Red asterisks indicate the five representative PB2 paired backgrounds selected for subsequent functional analysis.

Having identified six PB2 positions with distinct frequency patterns across epidemic waves and host groups, we examined the co-occurrence of their amino acid states in H7N9 sequences. All 15 possible pairwise combinations among positions 109, 122, 191, 340, 559, and 640 were analyzed separately in avian- and human-derived viruses across epidemic waves (Figure 2A,B). For each pair, prevalence was calculated using the number of sequences with unambiguous amino acid calls at both positions as the denominator. Detailed counts (x/n_pair) are provided in Supplementary Tables S2 and S3.

In avian-derived viruses, four paired backgrounds—109I + 191E, 191E + 640I, 109I + 640I, and 122A + 559T—were absent or uncommon during waves 1–3, increased in frequency during wave 4, remained detectable during wave 5, and became predominant after wave 5 (Figure 2A,C; Supplementary Table S3). In contrast, 191E + 340K increased in frequency through wave 5 but declined thereafter. These five paired backgrounds therefore showed two distinct temporal patterns in avian-derived viruses.

In human-derived viruses, 191E + 340K also increased through wave 5 and declined thereafter. The other four paired backgrounds remained uncommon during early waves and showed varying frequencies after wave 5 (Figure 2B,D; Supplementary Table S2). Only 11 human-derived sequences were available for the period after wave 5.

Based on these frequency patterns, five representative paired backgrounds—109I + 191E, 191E + 640I, 109I + 640I, 122A + 559T, and 191E + 340K—were selected for functional analysis. These included four pairs that became predominant in avian-derived viruses after wave 5 and one pair that peaked during wave 5. In subsequent experiments, the five pairs were introduced as substitutions into the early-wave JTC4 PB2 background: V109I + K191E, K191E + V640I, V109I + V640I, V122A + N559T, and K191E + R340K.

### 3.3. Representative PB2 Paired Substitutions Show Different Polymerase Activity Profiles in Human and Chicken Cells

**Figure 3:**
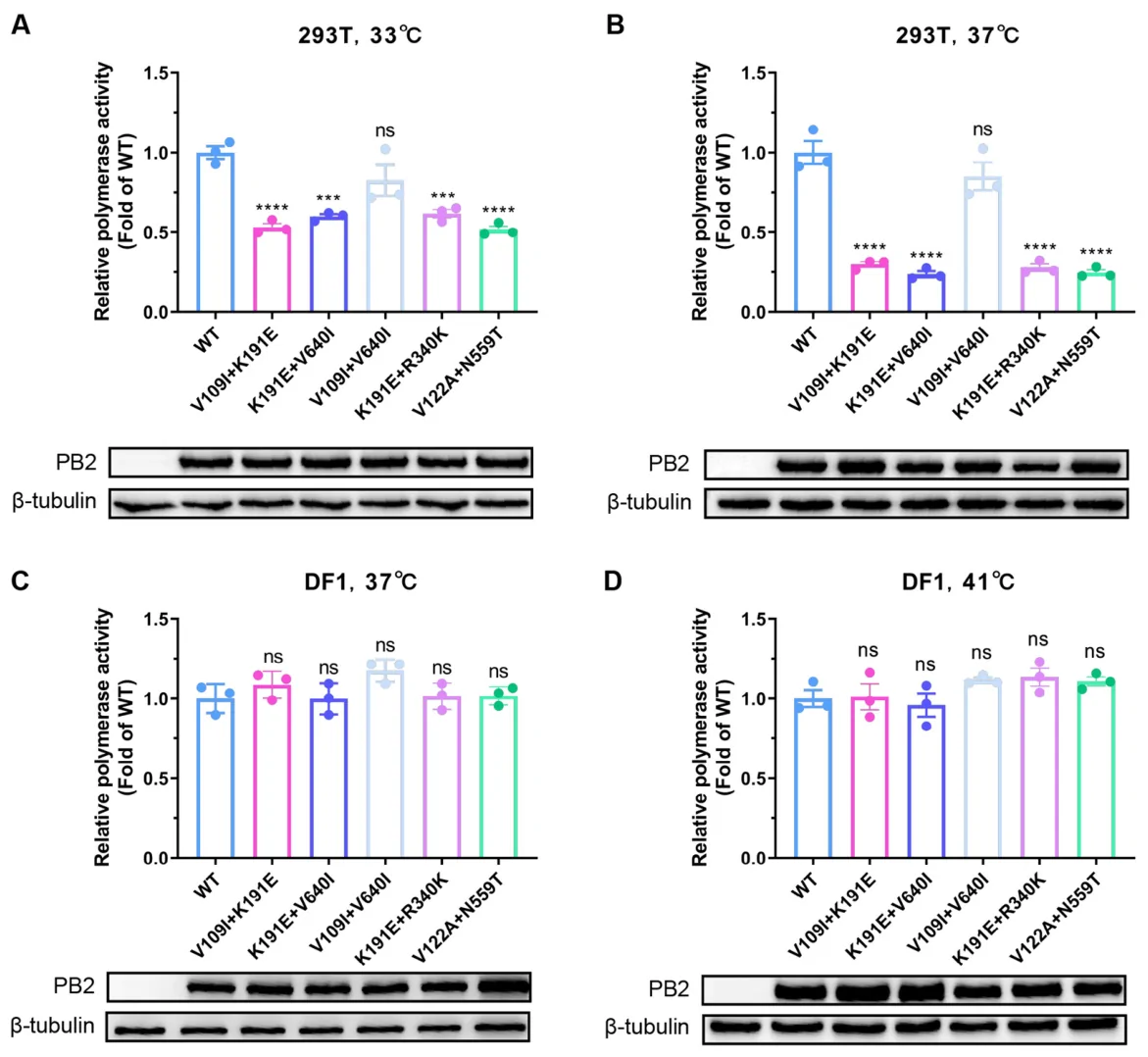
Effects of PB2 paired substitutions on polymerase activity in 293T and DF1 cells. (**A**–**D**) Minigenome polymerase activity of WT and five PB2 paired substitutions (V109I + K191E, K191E + V640I, V109I + V640I, K191E + R340K, and V122A + N559T) in 293T cells at 33 °C (**A**) and 37 °C (**B**), and DF1 cells at 37 °C (**C**) and 41 °C (**D**). Activity was normalized to WT under each condition. Corresponding PB2 and β-tubulin immunoblots are shown below the plots. Data are presented as mean ± SD from three independent experiments. Statistical analysis was performed using one-way ANOVA followed by Dunnett’s test versus WT. ns, not significant; *** *p* < 0.001; **** *p* < 0.0001.

To examine the functional effects of the five representative PB2 paired substitutions, we introduced them into the polymerase background of the early-wave H7N9 strain A/chicken/Eastern China/JTC4/2013. Polymerase activity was measured using minigenome assays in human 293T cells at 33 °C and 37 °C and in chicken DF1 cells at 37 °C and 41 °C (Figure 3A–D).

In 293T cells, four paired substitutions—V109I + K191E, K191E + V640I, K191E + R340K, and V122A + N559T—significantly reduced polymerase activity relative to WT at both 33 °C and 37 °C (Figure 3A,B). In contrast, V109I + V640I did not significantly alter polymerase activity at either temperature.

In DF1 cells, none of the five paired substitutions significantly altered polymerase activity relative to WT at 37 °C or 41 °C (Figure 3C,D). Thus, four paired substitutions reduced polymerase activity in 293T cells, whereas no significant changes were detected in DF1 cells under the tested conditions. These results reveal distinct polymerase activity profiles between the two cell models.

### 3.4. Single and Paired PB2 Substitutions Show Different Polymerase Activity Patterns in Human Cells

**Figure 4:**
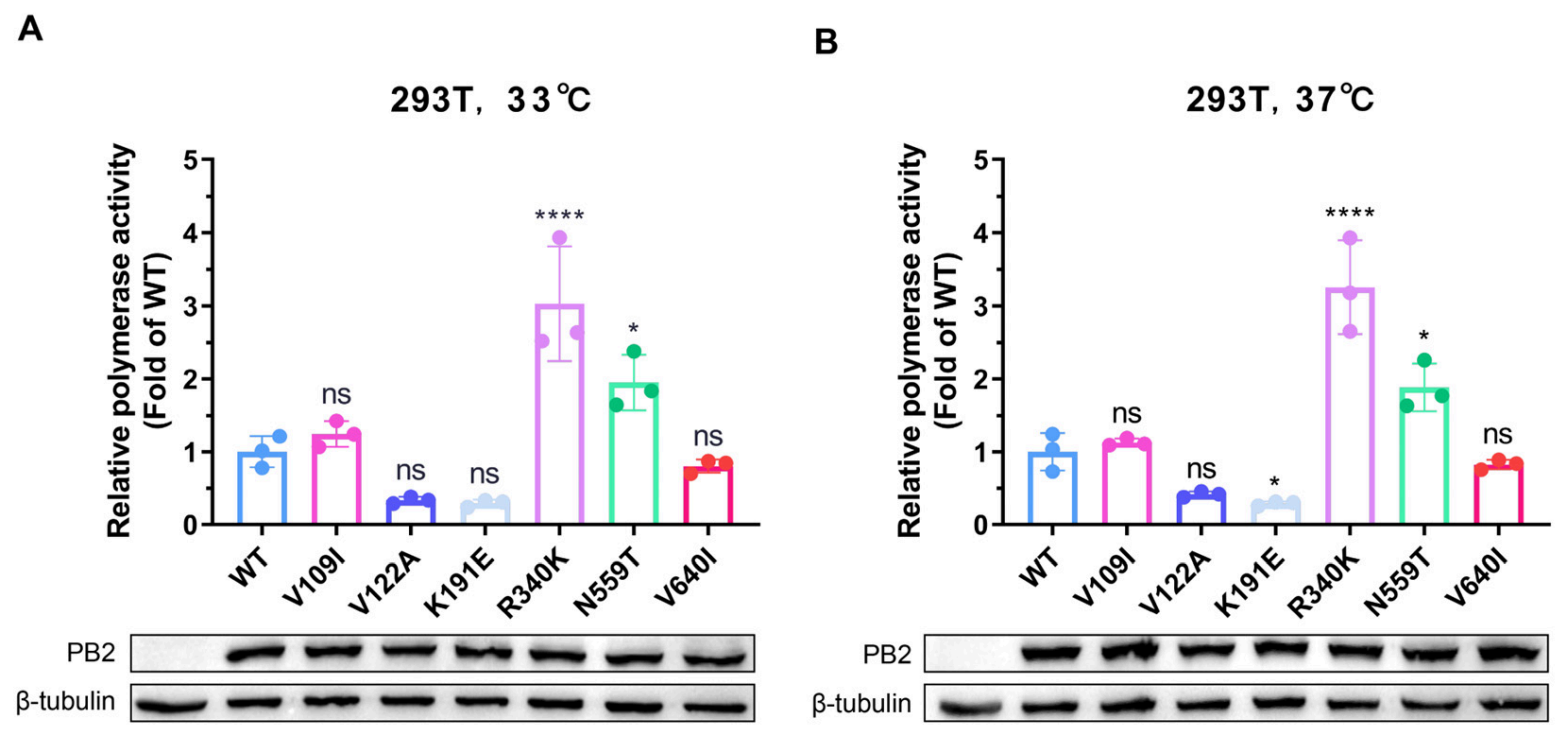
Effects of single PB2 substitutions on polymerase activity in human cells. (**A**,**B**) Minigenome polymerase activity of WT and six PB2 single substitutions (V109I, V122A, K191E, R340K, N559T, and V640I) in 293T cells at 33 °C (**A**) and 37 °C (**B**). Activity was normalized to WT under each condition. Corresponding PB2 and β-tubulin immunoblots are shown below the plots. Data are presented as mean ± SD from three independent experiments. Statistical analysis was performed using one-way ANOVA followed by Dunnett’s test versus WT. ns, not significant; * *p* < 0.05; **** *p* < 0.0001.

To compare the effects of individual PB2 substitutions with those of their paired combinations, six single substitutions (V109I, V122A, K191E, R340K, N559T, and V640I) were introduced separately into the same early-wave H7N9 PB2 background. Polymerase activity was measured in 293T cells at 33 °C and 37 °C using minigenome assays (Figure 4A,B).

The single substitutions showed different effects on polymerase activity at the two temperatures. At 33 °C, R340K markedly increased polymerase activity, while N559T produced a moderate but significant increase. V109I, V122A, K191E, and V640I did not significantly alter polymerase activity (Figure 4A). At 37 °C, R340K and N559T again increased polymerase activity, whereas K191E significantly reduced it. No significant changes were observed with V109I, V122A, or V640I (Figure 4B).

Comparison with the paired substitutions revealed different activity patterns. R340K alone increased polymerase activity, whereas K191E + R340K reduced activity relative to WT (Figure 3A,B and Figure 4A,B). Similarly, N559T increased activity when tested alone, while V122A + N559T reduced polymerase activity. V640I alone had no significant effect, whereas K191E + V640I reduced polymerase activity. These results show that several paired substitutions produced activity profiles that differed from those of their individual components.

### 3.5. Effects of ANP32 Overexpression on PB2 Paired-Substitution Polymerases in 293T Cells

**Figure 5:**
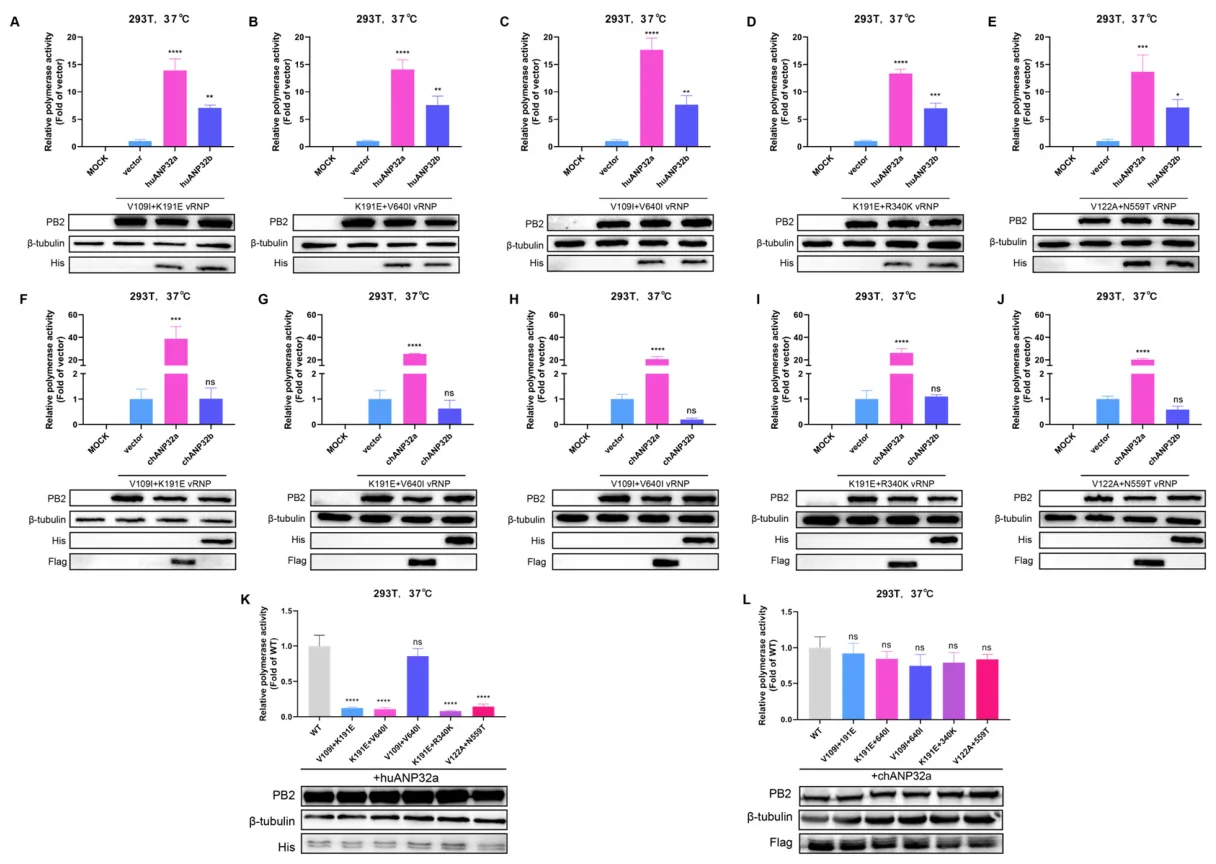
Effects of ANP32 overexpression on PB2 paired-substitution polymerase activity in 293T cells. (**A**–**E**) Effects of huANP32A and huANP32B on the indicated PB2 paired-substitution polymerases at 37 °C. (**F**–**J**) Effects of chANP32A and chANP32B on the same polymerases. (**K**,**L**) Polymerase activities of WT and paired substitutions under huANP32A (**K**) or chANP32A (**L**) overexpression. Activity was normalized to the corresponding empty-vector control (**A**–**J**) or WT (**K**,**L**). Mock served as a background control. Corresponding PB2, tagged ANP32, and β-tubulin immunoblots are shown below the plots. Data are presented as mean ± SD from three independent experiments. Statistical analysis was performed using one-way ANOVA followed by Dunnett’s test versus Vector (**A**–**J**) or WT (**K**,**L**). ns, not significant; * *p* < 0.05; ** *p* < 0.01; *** *p* < 0.001; **** *p* < 0.0001.

To examine the effects of ANP32 proteins on PB2 paired-substitution polymerases, human ANP32A (huANP32A), human ANP32B (huANP32B), chicken ANP32A (chANP32A), or chicken ANP32B (chANP32B) was co-expressed with WT or paired-substitution polymerases in 293T cells. Polymerase activity was measured using minigenome assays at 37 °C (Figure 5).

Compared with the corresponding empty-vector controls, overexpression of huANP32A or huANP32B increased polymerase activity in all five paired-substitution backgrounds (Figure 5A–E). Overexpression of chANP32A also increased polymerase activity, whereas chANP32B produced little or no detectable enhancement (Figure 5F–J).

We next compared the activities of WT and paired-substitution polymerases under huANP32A or chANP32A overexpression. In the presence of huANP32A, V109I + K191E, K191E + V640I, K191E + R340K, and V122A + N559T showed significantly lower polymerase activity than WT, whereas V109I + V640I did not differ significantly from WT (Figure 5K). Under chANP32A overexpression, none of the five paired-substitution polymerases differed significantly from WT (Figure 5L).

These results show that ANP32 overexpression affects polymerase activity in 293T cells, with different responses observed among the tested ANP32 proteins and PB2 backgrounds.

### 3.6. Human ANP32 Overexpression Does Not Significantly Enhance PB2 Paired-Substitution Polymerase Activity in DF1 Cells

**Figure 6:**
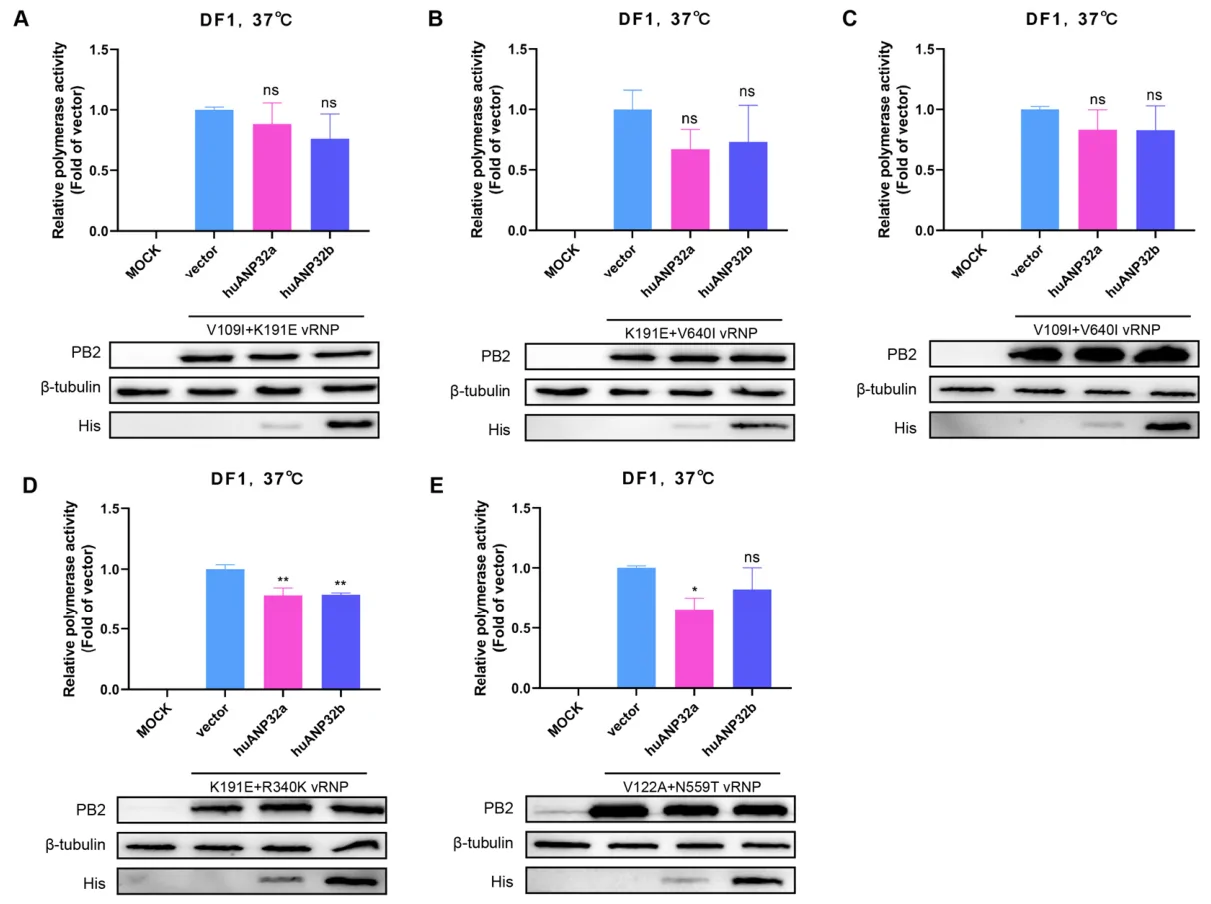
Effects of human ANP32 overexpression on PB2 paired-substitution polymerase activity in DF1 cells. (A–E) Minigenome polymerase activity of V109I + K191E (**A**), K191E + V640I (**B**), V109I + V640I (**C**), K191E + R340K (**D**), and V122A + N559T (**E**) in DF1 cells at 37 °C with mock, empty vector, huANP32A, or huANP32B. Activity was normalized to the corresponding empty-vector control. The MOCK group consisted of cells without plasmid transfection and served as an untransfected background control. Corresponding PB2, His-tagged ANP32, and β-tubulin immunoblots are shown below the plots. Data are presented as mean ± SD from three independent experiments. Statistical analysis was performed using one-way ANOVA followed by Dunnett’s test versus Vector. ns, not significant; * *p* < 0.05; ** *p* < 0.01.

To examine the effects of human ANP32 proteins in chicken cells, we expressed huANP32A or huANP32B with each of the five PB2 paired-substitution polymerases in DF1 cells. Polymerase activity was measured at 37 °C using minigenome assays (Figure 6A–E).

Neither huANP32A nor huANP32B significantly increased polymerase activity relative to the corresponding empty-vector control in any of the five paired-substitution backgrounds: V109I + K191E, K191E + V640I, V109I + V640I, K191E + R340K, or V122A + N559T (Figure 6A–E). In some backgrounds, polymerase activity was lower following huANP32A or huANP32B overexpression than with the vector control.

These results show that additional expression of human ANP32A or ANP32B did not significantly enhance the activity of the tested PB2 paired-substitution polymerases in DF1 cells under the conditions examined.

### 3.7. PB2 Paired Substitutions Do Not Abolish Detectable ANP32–PB2 Association

**Figure 7:**
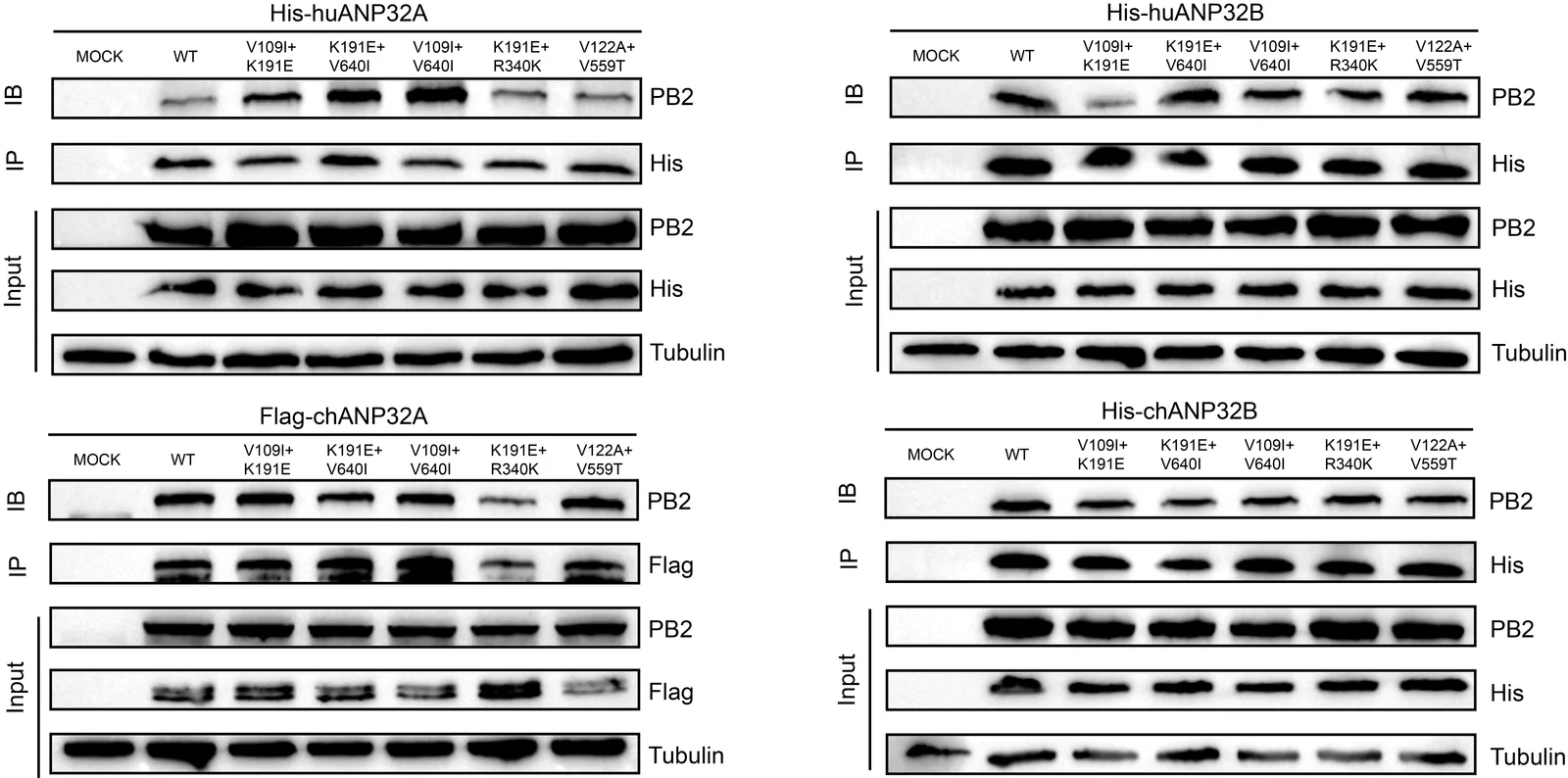
PB2 paired substitutions do not abolish detectable ANP32–PB2 interaction. Co-immunoprecipitation assays were performed in 293T cells by co-expressing tagged huANP32A, huANP32B, chANP32A, or chANP32B with WT PB2 or the indicated PB2 paired-substitution variants. ANP32 proteins were immunoprecipitated using anti-tag antibodies, and associated PB2 was detected by immunoblotting. Input and immunoprecipitated fractions are shown. PB2 remained detectable in ANP32 immunoprecipitates across WT and all tested paired-substitution backgrounds, indicating that these PB2 paired substitutions did not abolish detectable ANP32–PB2 interaction.

The ANP32 overexpression assays showed differences in polymerase activity among WT and PB2 paired-substitution backgrounds. To examine whether these differences were accompanied by a loss of detectable ANP32–PB2 association, we performed co-immunoprecipitation (Co-IP) assays in 293T cells expressing tagged human or chicken ANP32 proteins together with WT PB2 or the indicated paired-substitution variants (Figure 7).

PB2 was detected in ANP32 immunoprecipitates from cells expressing WT or any of the five paired variants: V109I + K191E, K191E + V640I, V109I + V640I, K191E + R340K, and V122A + N559T. This pattern was observed with huANP32A, huANP32B, chANP32A, and chANP32B (Figure 7).

### 3.8. Structural Modeling Shows Differences in Predicted PB2–ANP32A Interfaces

**Figure 8:**
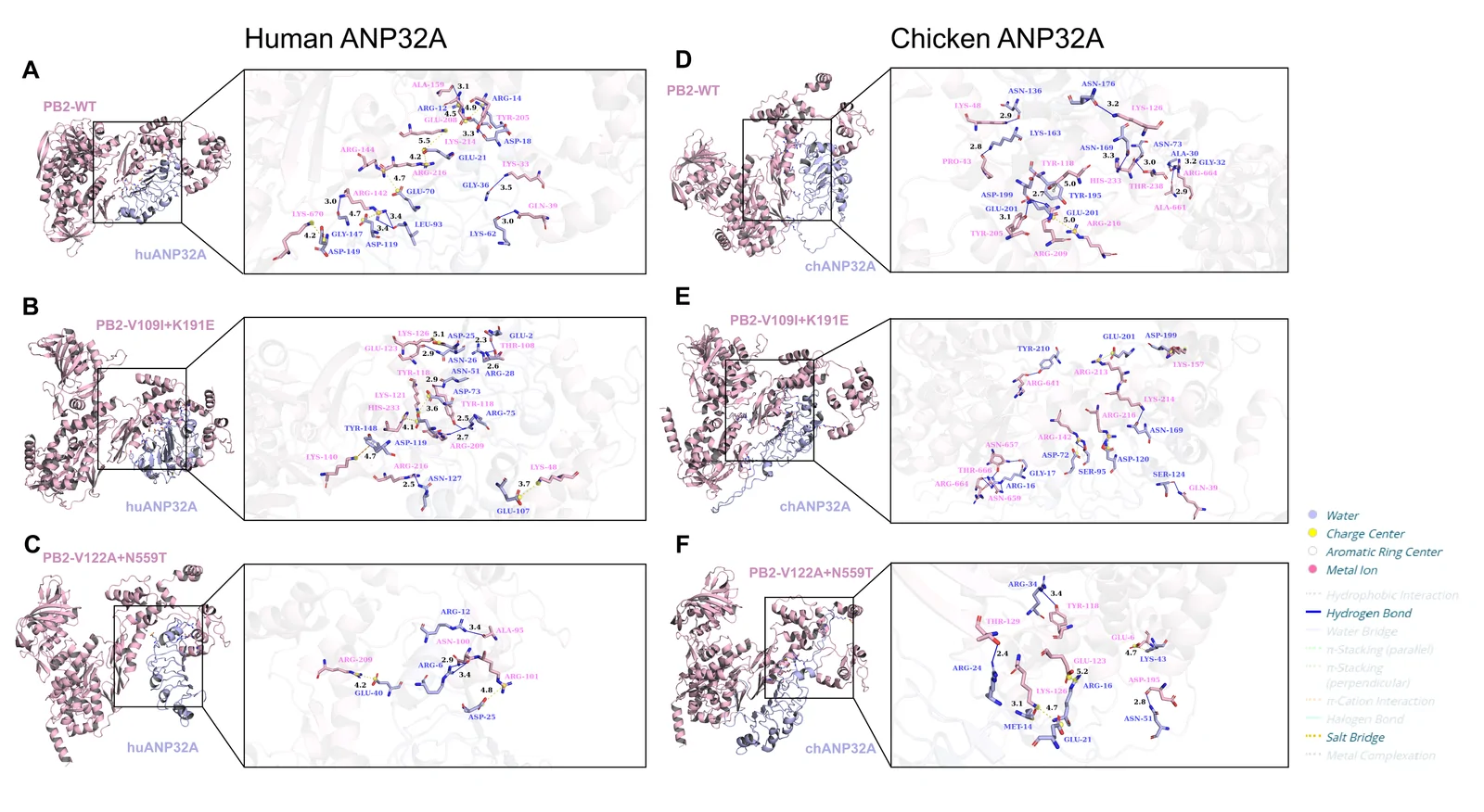
Predicted interaction interfaces between PB2 variants and human or chicken ANP32A. (**A**–**C**) Docking models of huANP32A with WT PB2 (**A**), V109I + K191E (**B**), and V122A + N559T (**C**). (**D**–**F**) Corresponding docking models with chANP32A. Predicted interface interactions were analyzed using PLIP and visualized using PyMOL.

To compare the predicted PB2–ANP32A interfaces, we modeled WT PB2 and two representative paired variants, V109I + K191E and V122A + N559T, in complex with huANP32A or chANP32A (Figure 8; Table 1). Both variants showed reduced polymerase activity in 293T cells, whereas neither differed significantly from WT in DF1 cells.

In the huANP32A models, the predicted WT interface contained multiple hydrogen bonds and electrostatic interactions. Compared with WT, V109I + K191E showed a different distribution of interface contacts, including hydrogen bonds, salt bridges, and cation–π interactions. Its HDOCK score was less negative than that of WT (−273.83 versus −286.96), whereas its MM/GBSA-estimated binding free energy was more negative (−66.97 versus −60.65 kcal/mol). V122A + N559T showed fewer predicted interface contacts, a less negative HDOCK score (−255.13), and a less negative estimated binding free energy (−50.41 kcal/mol) than WT (Figure 8A–C; Table 1).

In the chANP32A models, WT and both paired variants retained multiple predicted interface contacts, although their interaction patterns differed. The HDOCK scores were similar across the three models (−254.53 for WT, −258.50 for V109I + K191E, and −257.56 for V122A + N559T). However, the MM/GBSA-estimated binding free energies were less negative for V109I + K191E (−35.97 kcal/mol) and V122A + N559T (−39.86 kcal/mol) than for WT (−55.97 kcal/mol) (Figure 8D–F; Table 1).

Overall, structural modeling identified differences in the predicted PB2–ANP32A interface contacts between WT and the two paired variants. However, the docking scores and estimated binding free energies did not consistently correspond to the observed polymerase activities.

## 4. Discussion

This study identified recurrent PB2 paired backgrounds with distinct temporal patterns and polymerase activity profiles in human 293T and chicken DF1 cells. Four paired variants—V109I + K191E, K191E + V640I, K191E + R340K, and V122A + N559T—reduced polymerase activity in 293T cells at both tested temperatures, whereas none of the five variants differed significantly from WT in DF1 cells. In avian-derived sequences, four paired backgrounds increased in frequency after wave 5, while 191E + 340K peaked during wave 5 and subsequently declined. These results reveal variation in both the prevalence and functional profiles of naturally occurring PB2 combinations.

The single-substitution results highlight the importance of PB2 sequence context. R340K and N559T increased polymerase activity individually, whereas K191E + R340K and V122A + N559T reduced activity in 293T cells. At 37 °C, both K191E and K191E + V640I reduced polymerase activity, whereas V640I alone had no significant effect. Previous studies have described epistasis in influenza virus evolution [23] and the combined effects of substitutions across different H7N9 gene segments [24]. The detection of PB2-627K in chicken-derived H7N9 viruses [25] further illustrates the importance of interpreting individual markers within their viral backgrounds. Our findings extend this perspective to selected paired substitutions within PB2.

The ANP32 overexpression assays showed different responses across the tested cellular conditions. Previous studies have demonstrated species-specific differences in ANP32-mediated polymerase support, including the limited activity of chicken ANP32B [26]. Human ANP32A and ANP32B both contribute to human influenza A virus RNA replication [27]. In 293T cells, overexpression of huANP32A, huANP32B, or chANP32A increased polymerase activity relative to the corresponding vector controls, whereas chANP32B showed little or no enhancement. Under huANP32A overexpression, four paired variants had significantly lower activity than WT; under chANP32A overexpression, none differed significantly from WT. In DF1 cells, additional expression of huANP32A or huANP32B did not significantly enhance polymerase activity. These findings indicate that the response to ANP32 overexpression varies across the tested cellular conditions. Previous studies have characterized the polymerase–ANP32A interface [28] and the interaction between the ANP32 C-terminal acidic region and viral nucleoprotein [29], providing further context for these observations.

Co-immunoprecipitation detected PB2 in human and chicken ANP32 immunoprecipitates across WT and all five paired variants, despite differences in polymerase activity. ANP32A is involved in influenza replicase assembly [17], and structural studies have characterized ANP32-associated replication complexes [30] and H5N1 polymerase complexes containing ANP32B [31]. Specific ANP32A residues, including Asp149 and Asp152, also contribute to polymerase interactions [32].

Structural modeling revealed differences in the predicted PB2–ANP32A interfaces. In the huANP32A models, V109I + K191E had a less negative HDOCK score but a more negative MM/GBSA-estimated binding free energy than WT, despite its reduced polymerase activity in 293T cells. V122A + N559T showed fewer predicted interface contacts and a less negative estimated binding free energy. In the chANP32A models, both variants retained multiple predicted contacts but had less negative estimated binding free energies than WT. The predicted interface interactions and binding energies varied among PB2 variants but did not consistently correspond to their measured polymerase activities. The reported contribution of PB2 residue 473 to ANP32A-associated polymerase function [33] further highlights the relevance of PB2 sites beyond established adaptation markers.

The temporal patterns of these PB2 combinations occurred against a background of continued H7N9 evolution following poultry vaccination [34]. An antigenic variant was identified in 2019 [35], and subsequent studies documented the reintroduction of highly pathogenic H7N9 viruses into southwestern China [36] and continued genetic and antigenic differentiation [7]. Genomic epidemiological analysis also identified multiple H7N9 lineages circulating in Guangdong during wave 5 [37]. The increased frequency of four paired backgrounds after wave 5 may therefore reflect lineage expansion, linked substitutions elsewhere in the genome, reassortment, or differences in sequence sampling. Only 11 human-derived PB2 sequences were available after wave 5, further limiting comparisons between host groups during this period.

Overall, this study examined selected PB2 substitutions in a single early-wave H7N9 polymerase background using human 293T and chicken DF1 cells. The ANP32 assays were performed by overexpression, and the structural analyses were based on computational predictions.

## 5. Conclusions

Naturally occurring H7N9 PB2 paired backgrounds showed distinct temporal patterns and polymerase activity profiles in human 293T and chicken DF1 cells. Four of the five tested paired variants reduced polymerase activity relative to WT in 293T cells, whereas none differed significantly from WT in DF1 cells. Several paired variants also showed activity profiles distinct from those of their constituent single substitutions. Responses to ANP32 overexpression varied across the tested cellular conditions. These findings support considering paired PB2 substitutions alongside individual amino acid changes when evaluating H7N9 polymerase activity.

## Figures and Tables

**Table 1:** Docking scores, confidence scores, and MM/GBSA-estimated binding free energies of PB2–ANP32A complexes.

Complex	HDOCK Score	Confidence	Estimated Binding Free Energy (MM/GBSA; kcal/mol)
huANP32A–PB2 WT	−286.96	0.9393	−60.65
huANP32A–PB2 V109I + K191E	−273.83	0.9225	−66.97
huANP32A–PB2 V122A + N559T	−255.13	0.8912	−50.41
chANP32A–PB2 WT	−254.53	0.8900	−55.97
chANP32A–PB2 V109I + K191E	−258.50	0.8975	−35.97
chANP32A–PB2 V122A + N559T	−257.56	0.8957	−39.86

## Data Availability

The H7N9 PB2 sequences analyzed in this study were obtained from the GISAID EpiFlu database and are subject to its data access requirements. Aggregated sequence counts and pairwise prevalence data are presented in Supplementary Tables S1–S3.

## Supplementary Materials

The following supporting information can be downloaded at https://www.mdpi.com/article/10.3390/ani16213535/s1: Table S1: Dataset composition of H7N9 PB2 sequences used for wave- and host-stratified analysis; Table S2: Pairwise prevalence of PB2 residue-state combinations in human-derived H7N9 viruses; Table S3: Pairwise prevalence of PB2 residue-state combinations in avian-derived H7N9 viruses.

## Abbreviations

ANP32	Acidic nuclear phosphoprotein 32
PB2	Polymerase basic protein 2
PB1	Polymerase basic protein 1
PA	Polymerase acidic protein
NP	Nucleoprotein
WT	Wild type
GISAID	Global Initiative on Sharing All Influenza Data
DMEM	Dulbecco’s modified Eagle’s medium
FBS	Fetal bovine serum
SDS-PAGE	Sodium dodecyl sulfate–polyacrylamide gel electrophoresis
PVDF	Polyvinylidene fluoride
BSA	Bovine serum albumin
HRP	Horseradish peroxidase
IgG	Immunoglobulin G
ECL	Enhanced chemiluminescence
Co-IP	Co-immunoprecipitation
HDOCK	HDOCK protein–protein docking server
PLIP	Protein–Ligand Interaction Profiler
MM/GBSA	Molecular mechanics/generalized Born surface area
ANOVA	Analysis of variance

## References

[1] Gao R., Cao B., Hu Y., Feng Z., Wang D., Hu W., Chen J., Jie Z., Qiu H., Xu K. (2013). Human Infection with a Novel Avian-Origin Influenza a (H7N9) Virus. N. Engl. J. Med..

[2] Wang X., Jiang H., Wu P., Uyeki T.M., Feng L., Lai S., Wang L., Huo X., Xu K., Chen E. (2017). Epidemiology of avian influenza A H7N9 virus in human beings across five epidemics in mainland China, 2013–17: An epidemiological study of laboratory-confirmed case series. Lancet Infect. Dis..

[3] World Health Organization (2026). Human Infection with Avian Influenza A(H7N9) Virus in China. https://www.who.int/westernpacific/publications/m/item/avian-influenza-weekly-update---1052--10-july-2026.

[4] Zeng X., Tian G., Shi J., Deng G., Li C., Chen H. (2018). Vaccination of poultry successfully eliminated human infection with H7N9 virus in China. Sci. China Life Sci..

[5] Yin X., Deng G., Zeng X., Cui P., Hou Y., Liu Y., Fang J., Pan S., Wang D., Chen X. (2021). Genetic and biological properties of H7N9 avian influenza viruses detected after application of the H7N9 poultry vaccine in China. PLoS Pathog..

[6] Wu Y., Hu J., Jin X., Li X., Wang J., Zhang M., Chen J., Xie S., Qi W., Liao M. (2021). Accelerated Evolution of H7N9 Subtype Influenza Virus under Vaccination Pressure. Virol. Sin..

[7] Liu Y., Chen Y., Yang Z., Lin Y., Fu S., Chen J., Xu L., Liu T., Niu B., Huang Q. (2024). Evolution and Antigenic Differentiation of Avian Influenza A(H7N9) Virus, China. Emerg. Infect. Dis..

[8] Mok C.K., Lee H.H., Lestra M., Nicholls J.M., Chan M.C., Sia S.F., Zhu H., Poon L.L., Guan Y., Peiris J.S. (2014). Amino acid substitutions in polymerase basic protein 2 gene contribute to the pathogenicity of the novel A/H7N9 influenza virus in mammalian hosts. J. Virol..

[9] Liu W.J., Li J., Zou R., Pan J., Jin T., Li L., Liu P., Zhao Y., Yu X., Wang H. (2020). Dynamic PB2-E627K substitution of influenza H7N9 virus indicates the in vivo genetic tuning and rapid host adaptation. Proc. Natl. Acad. Sci. USA.

[10] Soh Y.Q.S., Moncla L.H., Eguia R., Bedford T., Bloom J.D. (2019). Comprehensive mapping of adaptation of the avian influenza polymerase protein PB2 to humans. eLife.

[11] Pauly M.D., Lyons D.M., Fitzsimmons W.J., Lauring A.S. (2017). Epistatic Interactions within the Influenza A Virus Polymerase Complex Mediate Mutagen Resistance and Replication Fidelity. mSphere.

[12] Zhou Y., Wu X., Yan D., Chen C., Liu X., Huang C., Fu X., Tian G., Ding C., Wu J. (2021). V292I mutation in PB2 polymerase induces increased effects of E627K on influenza H7N9 virus replication in cells. Virus Res..

[13] Wang X., Zheng H., Gao R., Ren L., Jin M., Ji Z., Wang X., Lu X., Yang W., Gu M. (2023). Genetically Related Avian Influenza H7N9 Viruses Exhibit Different Pathogenicity in Mice. Animals.

[14] Wang X., Tang X.E., Zheng H., Gao R., Lu X., Yang W., Zhou L., Chen Y., Gu M., Hu J. (2024). Amino acid mutations PB1-V719M and PA-N444D combined with PB2-627K contribute to the pathogenicity of H7N9 in mice. Vet. Res..

[15] Long J.S., Giotis E.S., Moncorgé O., Frise R., Mistry B., James J., Morisson M., Iqbal M., Vignal A., Skinner M.A. (2016). Species difference in ANP32A underlies influenza A virus polymerase host restriction. Nature.

[16] Staller E., Sheppard C.M., Neasham P.J., Mistry B., Peacock T.P., Goldhill D.H., Long J.S., Barclay W.S. (2019). ANP32 Proteins Are Essential for Influenza Virus Replication in Human Cells. J. Virol..

[17] Carrique L., Fan H., Walker A.P., Keown J.R., Sharps J., Staller E., Barclay W.S., Fodor E., Grimes J.M. (2020). Host ANP32A mediates the assembly of the influenza virus replicase. Nature.

[18] Peacock T.P., Sheppard C.M., Lister M.G., Staller E., Frise R., Swann O.C., Goldhill D.H., Long J.S., Barclay W.S. (2023). Mammalian ANP32A and ANP32B Proteins Drive Differential Polymerase Adaptations in Avian Influenza Virus. J. Virol..

[19] Yan Y., Zhang D., Zhou P., Li B., Huang S.Y. (2017). HDOCK: A web server for protein-protein and protein-DNA/RNA docking based on a hybrid strategy. Nucleic Acids Res..

[20] Yan Y., Tao H., He J., Huang S.Y. (2020). The HDOCK server for integrated protein-protein docking. Nat. Protoc..

[21] Weng G., Wang E., Wang Z., Liu H., Zhu F., Li D., Hou T. (2019). HawkDock: A web server to predict and analyze the protein-protein complex based on computational docking and MM/GBSA. Nucleic Acids Res..

[22] Schake P., Bolz S.N., Linnemann K., Schroeder M. (2025). PLIP 2025: Introducing Protein–Protein Interactions to the Protein–Ligand Interaction Profiler. Nucleic Acids Res..

[23] Lyons D.M., Lauring A.S. (2018). Mutation and Epistasis in Influenza Virus Evolution. Viruses.

[24] Zheng H., Chen Y., Gao R., Yang W., Lu X., Chen Y., Gu M., Hu J., Liu X., Hu S. (2026). NA-N322S and G389D mutations synergize with PB2-627K to enhance H7N9 pathogenicity in mammals. Vet. Microbiol..

[25] Gu J., Gu M., Yan Y., Liu K., Wang X., Xu X., Liu X. (2020). Detection of PB2 627 K mutation in two highly pathogenic isolates of the H7N9 subtype Influenza a virus from chickens in Northern China. J. Infect..

[26] Long J.S., Idoko-Akoh A., Mistry B., Goldhill D., Staller E., Schreyer J., Ross C., Goodbourn S., Shelton H., Skinner M.A. (2019). Species specific differences in use of ANP32 proteins by influenza A virus. eLife.

[27] Zhang H., Zhang Z., Wang Y., Wang M., Wang X., Zhang X., Ji S., Du C., Chen H., Wang X. (2019). Fundamental Contribution and Host Range Determination of ANP32A and ANP32B in Influenza A Virus Polymerase Activity. J. Virol..

[28] Mistry B., Long J.S., Schreyer J., Staller E., Sanchez-David R.Y., Barclay W.S. (2020). Elucidating the Interactions between Influenza Virus Polymerase and Host Factor ANP32A. J. Virol..

[29] Wang F., Sheppard C.M., Mistry B., Staller E., Barclay W.S., Grimes J.M., Fodor E., Fan H. (2022). The C-terminal LCAR of host ANP32 proteins interacts with the influenza A virus nucleoprotein to promote the replication of the viral RNA genome. Nucleic Acids Res..

[30] Arragain B., Krischuns T., Pelosse M., Drncova P., Blackledge M., Naffakh N., Cusack S. (2024). Structures of influenza A and B replication complexes give insight into avian to human host adaptation and reveal a role of ANP32 as an electrostatic chaperone for the apo-polymerase. Nat. Commun..

[31] Staller E., Carrique L., Swann O.C., Fan H., Keown J.R., Sheppard C.M., Barclay W.S., Grimes J.M., Fodor E. (2024). Structures of H5N1 influenza polymerase with ANP32B reveal mechanisms of genome replication and host adaptation. Nat. Commun..

[32] Park Y.H., Woo S.J., Chungu K., Lee S.B., Shim J.H., Lee H.J., Kim I., Rengaraj D., Song C.S., Suh J.Y. (2021). Asp149 and Asp152 in chicken and human ANP32A play an essential role in the interaction with influenza viral polymerase. FASEB J..

[33] Zhang M., Liu M., Chen H., Qiu T., Jin X., Fu W., Teng Q., Zhao C., Xu J., Li Z. (2024). PB2 residue 473 contributes to the mammalian virulence of H7N9 avian influenza virus by modulating viral polymerase activity via ANP32A. J. Virol..

[34] Hou Y., Deng G., Cui P., Zeng X., Li B., Wang D., He X., Yan C., Zhang Y., Li J. (2024). Evolution of H7N9 highly pathogenic avian influenza virus in the context of vaccination. Emerg. Microbes Infect..

[35] Jiang W., Hou G., Li J., Peng C., Wang S., Liu S., Zhuang Q., Yuan L., Yu X., Li Y. (2020). Antigenic Variant of Highly Pathogenic Avian Influenza A(H7N9) Virus, China, 2019. Emerg. Infect. Dis..

[36] He D., Gu M., Wang X., Yan Y., Li Y., Wang X., Hu S., Liu X. (2023). Reintroduction of highly pathogenic avian influenza A H7N9 virus in southwestern China. Virus Genes.

[37] Bai R., Sikkema R.S., Munnink B.B.O., Li C.R., Wu J., Zou L., Jing Y., Lu J., Yuan R.Y., Liao M. (2020). Exploring utility of genomic epidemiology to trace origins of highly pathogenic influenza A/H7N9 in Guangdong. Virus Evol..

