# Supplementary material for: Naturally Occurring PB2 Paired Substitutions Confer Host-Dependent Polymerase Phenotypes in H7N9 Avian Influenza Viruses with Differential ANP32 Support
Source: Animals (Basel). 2026 Nov 9;16(21):3535. doi: 10.3390/ani16213535 (PMC13762209; doi:10.3390/ani16213535)
Supplement: Supplementary file 1 [file animals-16-03535-s001.zip › animals-4598205-supplementary.pdf]

Supplementary Materials

Supplementary Table S1. Dataset composition of H7N9 PB2 sequences used for wave- and host-stratified analysis.

H7N9 PB2 sequences obtained from GISAID were grouped by host origin and epidemic wave. After removal of duplicate accession records, the dataset comprised 899 avian-derived and 1,087 human-derived sequences. Epidemic-wave definitions are provided in Section 2.1.

Host	W1	W2	W3	W4	W5	After W5	Total
Date range	Feb 2013–Sep 2013	Oct 2013–Sep 2014	Oct 2014–Sep 2015	Oct 2015–Sep 2016	Oct 2016–Sep 2017	Oct 2017–Dec 2025	
Avian-derived	72	399	110	60	198	60	899
Human-derived	129	267	156	45	479	11	1087
Total	201	666	266	105	677	71	1986

Supplementary Table S2. Pairwise prevalence of PB2 residue-state combinations in human-derived H7N9 viruses.

All 15 pairs among PB2 residue states 109I, 122A, 191E, 340K, 559T, and 640I were analyzed across epidemic waves. Values are shown as x/n_pair (%), where x is the number of sequences carrying both indicated residues and n_pair is the number with unambiguous amino acid calls at both positions. The post-wave-5 group contained 11 sequences.

Paired background	W1	W2	W3	W4	W5	After W5
109I+122A	0/122(0.00%)	0/266(0.00%)	0/151(0.00%)	17/45(37.78%)	89/478(18.62%)	10/11(90.91%)
109I+191E	0/121(0.00%)	0/262(0.00%)	0/150(0.00%)	17/45(37.78%)	90/477(18.87%)	2/11(18.18%)
109I+340K	0/120(0.00%)	0/261(0.00%)	0/151(0.00%)	0/44(0.00%)	2/478(0.42%)	0/11(0.00%)
109I+559T	0/120(0.00%)	0/265(0.00%)	0/150(0.00%)	17/45(37.78%)	90/477(18.87%)	10/11(90.91%)
109I+640I	0/122(0.00%)	0/266(0.00%)	0/150(0.00%)	17/45(37.78%)	88/477(18.45%)	10/11(90.91%)
122A+191E	0/121(0.00%)	0/262(0.00%)	0/150(0.00%)	20/45(44.44%)	91/478(19.04%)	2/11(18.18%)
122A+340K	0/120(0.00%)	0/261(0.00%)	0/151(0.00%)	0/44(0.00%)	2/479(0.42%)	0/11(0.00%)
122A+559T	0/120(0.00%)	0/265(0.00%)	0/150(0.00%)	20/45(44.44%)	91/478(19.04%)	10/11(90.91%)
122A+640I	0/121(0.00%)	0/266(0.00%)	0/150(0.00%)	20/45(44.44%)	89/478(18.62%)	10/11(90.91%)
191E+340K	1/120(0.83%)	7/260(2.69%)	28/153(18.30%)	21/44(47.73%)	382/478(79.92%)	1/11(9.09%)
191E+559T	8/120(6.67%)	243/261(93.10%)	139/152(91.45%)	44/45(97.78%)	468/477(98.11%)	3/11(27.27%)
191E+640I	0/120(0.00%)	0/262(0.00%)	2/152(1.32%)	20/45(44.44%)	88/477(18.45%)	2/11(18.18%)
340K+559T	1/120(0.83%)	6/260(2.31%)	33/154(21.43%)	21/44(47.73%)	381/478(79.71%)	1/11(9.09%)
340K+640I	0/120(0.00%)	0/261(0.00%)	0/154(0.00%)	0/44(0.00%)	0/478(0.00%)	0/11(0.00%)
559T+640I	0/120(0.00%)	0/266(0.00%)	2/154(1.30%)	20/45(44.44%)	89/478(18.62%)	10/11(90.91%)

Supplementary Table S3. Pairwise prevalence of PB2 residue-state combinations in avian-derived H7N9 viruses.

All 15 pairs among PB2 residue states 109I, 122A, 191E, 340K, 559T, and 640I were analyzed across epidemic waves. Values are shown as x/n_pair (%), where x is the number of sequences carrying both indicated residues and n_pair is the number with unambiguous amino acid calls at both positions.

Paired background	W1	W2	W3	W4	W5	After W5
109I+122A	0/72(0.00%)	0/397(0.00%)	0/110(0.00%)	17/60(28.33%)	56/198(28.28%)	47/60(78.33%)
109I+191E	0/72(0.00%)	0/399(0.00%)	0/110(0.00%)	18/60(30.00%)	58/198(29.29%)	45/60(75.00%)
109I+340K	0/72(0.00%)	0/394(0.00%)	0/110(0.00%)	0/60(0.00%)	1/198(0.51%)	3/60(5.00%)
109I+559T	0/72(0.00%)	0/393(0.00%)	0/110(0.00%)	18/60(30.00%)	57/198(28.79%)	48/60(80.00%)
109I+640I	0/72(0.00%)	0/393(0.00%)	0/110(0.00%)	16/60(26.67%)	57/198(28.79%)	47/60(78.33%)
122A+191E	0/72(0.00%)	0/397(0.00%)	0/110(0.00%)	19/60(31.67%)	58/198(29.29%)	54/60(90.00%)
122A+340K	0/72(0.00%)	0/392(0.00%)	0/110(0.00%)	1/60(1.67%)	0/198(0.00%)	3/60(5.00%)
122A+559T	0/72(0.00%)	0/391(0.00%)	0/110(0.00%)	19/60(31.67%)	57/198(28.79%)	53/60(88.33%)
122A+640I	0/72(0.00%)	0/391(0.00%)	0/110(0.00%)	17/60(28.33%)	58/198(29.29%)	52/60(86.67%)
191E+340K	0/72(0.00%)	5/394(1.27%)	27/110(24.55%)	26/60(43.33%)	135/198(68.18%)	3/60(5.00%)
191E+559T	11/72(15.28%)	386/393(98.22%)	101/110(91.82%)	58/60(96.67%)	192/198(96.97%)	53/60(88.33%)
191E+640I	0/72(0.00%)	0/393(0.00%)	1/110(0.91%)	17/60(28.33%)	59/198(29.80%)	50/60(83.33%)
340K+559T	0/72(0.00%)	3/393(0.76%)	29/110(26.36%)	25/60(41.67%)	130/198(65.66%)	3/60(5.00%)
340K+640I	0/72(0.00%)	0/393(0.00%)	1/110(0.91%)	0/60(0.00%)	0/198(0.00%)	3/60(5.00%)
559T+640I	0/72(0.00%)	0/393(0.00%)	1/110(0.91%)	17/60(28.33%)	58/198(29.29%)	53/60(88.33%)
